# Equine metabolic syndrome impairs adipose stem cells osteogenic differentiation by predominance of autophagy over selective mitophagy

**DOI:** 10.1111/jcmm.12932

**Published:** 2016-09-14

**Authors:** Krzysztof Marycz, Katarzyna Kornicka, Monika Marędziak, Paweł Golonka, Jakub Nicpoń

**Affiliations:** ^1^ Electron Microscopy LaboratoryThe Faculty of Biology and Animal ScienceUniversity of Environmental and Life Sciences WroclawWroclawPoland; ^2^ Wroclaw Research Centre EIT+WrocławPoland; ^3^ Department of Animal Physiology and BiostructureFaculty of Veterinary MedicineUniversity of Environmental and Life Sciences WroclawWroclawPoland; ^4^ Equine Clinic EquivetGliwicePoland; ^5^ Department of SurgeryFaculty of Veterinary MedicineUniversity of Environmental and Life Sciences WroclawWroclawPoland

**Keywords:** mitochondria, autophagy, mitophagy, mitochondria biogenesis, equine metabolic syndrome, adipose derived stem cells

## Abstract

Adipose‐derived mesenchymal stem cells (ASC) hold great promise in the treatment of many disorders including musculoskeletal system, cardiovascular and/or endocrine diseases. However, the cytophysiological condition of cells, used for engraftment seems to be fundamental factor that might determine the effectiveness of clinical therapy. In this study we investigated growth kinetics, senescence, accumulation of oxidative stress factors, mitochondrial biogenesis, autophagy and osteogenic differentiation potential of ASC isolated from horses suffered from equine metabolic syndrome (EMS). We demonstrated that EMS condition impairs multipotency/pluripotency in ASCs causes accumulation of reactive oxygen species and mitochondria deterioration. We found that, cytochrome c is released from mitochondria to the cytoplasm suggesting activation of intrinsic apoptotic pathway in those cells. Moreover, we observed up‐regulation of p21 and decreased ratio of Bcl‐2/BAX. Deteriorations in mitochondria structure caused alternations in osteogenic differentiation of ASC_EMS_ resulting in their decreased proliferation rate and reduced expression of osteogenic markers BMP‐2 and collagen type I. During osteogenic differentiation of ASC_EMS_, we observed autophagic turnover as probably, an alternative way to generate adenosine triphosphate and amino acids required to increased protein synthesis during differentiation. Downregulation of PGC1α, PARKIN and PDK4 in differentiated ASC_EMS_ confirmed impairments in mitochondrial biogenesis and function. Hence, application of ASC_EMS_ into endocrinological or ortophedical practice requires further investigation and analysis in the context of safeness of their application.

## Introduction

Stem cells hold globally great promise in the treatment of many disorders including musculoskeletal system, cardiovascular and/or endocrine diseases. Recently, endocrine disorders have become a growing problem in both human as well as veterinary medicine 1, 2, 3, 4, 5. In recent years more and more horses worldwide suffer for equine metabolic syndrome (EMS), due to high starch, high carbohydrates diet (rich in cereals) and elevated environmental stress, that lead to excessive cortisol production and free radicals production 6, 7, 8. Equine metabolic syndrome culminates with laminitis, which exclude horses from their further activity, and becomes clinical disorder with serious health and social consequences, that often still requires euthanasia. Recently, data indicate on beneficial/promising effects of mesenchymal stem progenitor cells (MSC) in the course of diabetes type II treatment in rodents 9, 10. Mesenchymal stem progenitor cell harvested from adipose tissue (ASC adipose derived mesenchymal stromal stem cells) has been showed to improved metabolic control, decreased insulin requirements, ameliorate insulin sensitivity and increased islets numbers in the pancreas 11, 12. However, a key factor, which determines the efficiency and effectiveness of cellular therapy is physiological condition of engrafted cells.

Adipose derived mesenchymal stem cells are characterized by multi‐lineage differentiation potential and what is more the ability to regulate immune response 13, 14, 15. They exhibit the presence of specific surface markers including CD90^+^, CD105^+^, CD44^+^ and lack expression of CD45^−^, elevated proliferative potential, viability, ability to self‐renew and clonogenic potential (CFU‐fs)^16^, ^17^. These features allow ASC to fulfil their multiple functions, namely to control tissue homeostasis and to ensure regeneration and tissue repair 18. The maintenance of self‐renew and proliferative potential in ASC are regulate by the expression of transcription factors including Octamer Binding Transcription Factor‐4 (Oct4), Sex Determining Region Y Box‐2 (SOX2) and Nanog Homeobox Protein (Nanog). It is also believe, that telomerase reverse transcriptase (TERT), that is up‐regulated in the rapidly dividing adult stem cells, are responsible for regulation of lifespan by allowing for indefinite division without shortening of telomeres 19, 20, 21. Consequently, Oct4/Sox‐2/Nanog/TERT are tandem genes that maintained multipotent/pluripotent character of ACS, however abbreviation in their activity, caused for example by insulin resistance or over expression of pro‐inflammatory cytokines *i.e*. IL‐6 might seriously reduce ASC stemness. Besides multipotency, ASC exhibits immunomodulatory and immunosuppressive properties, which make them even more promising tool in cellular therapies 15, 22, 23, especially in the context of their potential clinical application in EMS treatment.

One of the explanations of the regenerative ability of ASC is their paracrine action through secretion of membrane derived vesicles (MVs), which contain a wide range of growth factors, including bone morphogenetic protein (BMP‐2), VEGF, fibroblast growth factor 24, 25. Moreover, MVs also contain mRNA, as well as anti‐apoptotic and anti‐inflammatory factors such as interleukin 10 (IL‐10), transforming growth factor beta, IL‐4 and IL‐13 26, 27, 28. Finally, ASC possess ability to differentiation *in vitro* into multiple cell lineages including osteocytes, chondrocytes, myocytes and neurons 29, 30. These features seem to be crucial, from clinical perspective, especially, that in the last decade equine cellular therapies in treatment of various musculoskeletal disorders are widely applied in veterinary clinical practice 31, 32, 33.

However, the impact of progressive oxidative stress, apoptosis, mitochondrial function deterioration as well as elevated senescence and ageing, that are characteristic for EMS derived ASC 34 in the context of their influence on osteogenic differentiation are still not fully described. Both oxidative stress and epigenetic modifications of the genome are recognized as crucial factors initiating ageing and senescence in MSC that in consequence might seriously impairs their osteogenic differentiation potential 35. Our previous data suggest that the elevated accumulation of stress factors in ASC isolated from EMS horses, including reactive oxygen species (ROS) and nitric oxide, might be the main reason of the their cytological impairment 34. The increase in the ROS and nitric oxide content simultaneously with decreased anti‐oxidative protection coming from superoxide dismutase (SOD), lead to permanent growth arrest or apoptosis, which are initiated by up‐regulation of p21, p53 (tumour suppressor) and BAX expression, cytochrome C relocation, chromatin condensation and remodelling 36.

Autophagy is the mechanism, that protects cells against cellular damage, extracellular stress conditions (nutrient deprivation, hypoxia, oxidative stress), intracellular stress conditions (endoplasmic reticulum stress, accumulation of damaged organelles and aggregation of proteins) and/or finally apoptosis 37. In the course of these process, the autophagosomes absorbed damaged cellular components and transferred them to lysosomes, where recycling of nutrients and/or constituents have been observed. These mechanism was widely described in the many various disease such as cancer, infectious diseases, neurodegenerative disorders and finally diabetes type II 38, 39. The large number of stimuli, that are able to trigger autophagy, implies the involvement of multiple signalling pathways in autophagosome formation. The autophagy is controlled and regulated by autophagy‐related genes and their products called ATG and Atg respectively. In the process of initiation of autophagosome formation during autophagy, Beclin 1 through interacts with class III PI3K are recognized as a central player. Beclin 1 has been also shown to stimulate autophagy in cancer cells, and may be potent autophagy‐regulating targets for genetic intervention. Autophagy, as a dynamic process, might be broken down into few steps including: (*i*) induction, (*ii*) autophagosome formation, (*iii*) autophagolysosome formation and (*iv*) delivery and degradation of the autophagic body. However, what is worth nothing, initiation of autophagy *per se*, is not always associated with autolysosome formation and does not necessarily lead to apoptosis. It seems that autophagy in progenitor stromal stem cells, that exhibit self‐renew properties might be temporary self‐defense process that protects cells from unfavourable environmental factors and might lead to cells quiescence. On the other hand, pro‐inflammatory and oxidative micro‐environmental of adipose tissue in diabetic condition 6 might be an stimuli factor for progenitor cells to induce and enhance their multipotency through autophagy. Interestingly, it was demonstrated that autophagy prevents β‐cell apoptosis and improves ER stress‐induced diabetes mice 40. Recent evidence indicate that MSCs stimulated autophagy in neural cell line, that in consequence lead to increased neuronal survival in neurodegenerative disorders 41. However, the cyto‐physiological balance between mitophagy, mitochondrial biogenesis and ‘stemness’ seems to be crucial correlated factors in the maintenance of cell energetic status, which allows progenitors to undertake a specific cellular functions. Moreover, recently it was demonstrated, that infused bone marrow derived MSCs in streptozotocin‐induced (STZ‐induced) type 2 diabetic rat, promote formation of autophagosomes and autolysosomes and thus lead to improvement of β‐cell function and survival 40. Selective autophagy of mitochondria, known as mitophagy, is an important mitochondrial quality control mechanism that eliminates damaged mitochondria and thus might be recognized as an efficient cellular protective process. The elimination of damaged mitochondria is mediated by a pathway comprised of PTEN‐induced putative protein kinase 1 and the E3 ubiquitin ligase Parkin. They accumulate on damaged mitochondria, promote their segregation from the mitochondrial network, and target these organelles for autophagic degradation in a process that requires Parkin‐dependent ubiquitination of mitochondrial proteins 42. Ensuring proper elimination of dysfunctional mitochondria that has been implicated in EMS derived progenitor cells may be essential for cellular survival.

Thus, we were interested weather ASC derived from EMS horses (ASC_EMS_), that are affected by elevated apoptosis, senesce, mitochondrial dysfunctions and finally multipotency impairment are protected by the induction of selective mitophagy in the both native and osteogenic differentiation conditions. Here, we demonstrated, that EMS condition impairs multipotency/pluripotency in ASCs, causes imperfections in the formation of extracellular matrix through downregulation of BMP‐2 and Coll‐1. Moreover, we have found, that ASC obtained from EMS horses displayed reduced mitochondrial biogenesis, however autophagosomes and autolysosomes formation was increased as a biological attempt to maintenance ‘stemness’. Still, more data are required to investigate and understand the effect of EMS condition on adipose tissue progenitor stem cells in the context of their potential clinical application in autologous treatment of EMS.

## Materials and methods

All reagents used in this experiment were purchased from Sigma‐Aldrich (Poznan Poland), unless indicated otherwise.

### Animals qualification

All experimental procedures were approved by the II Local Ethics Committee of Environmental and Life Sciences University (Chelmonskiego 38C, 51‐630 Wroclaw, Poland; decision No. 84/2012). Procedures were performed under local anaesthesia using 2% lignocaine (Polfa S.A., Warsaw, Poland). All horses were age‐matched (mixed sex, 9–14 years; mean ± S.D., 11.2 ± 1.7 years) and divided into two groups: EMS group (*n* = 6) and control, healthy horses (*n* = 6). Table 1 shows detailed characterization of animals used in this study. Qualification to the experimental groups was performed based on (*i*) extensive interviews with owners, (*ii*) measurement of bodyweight, (*iii*) estimation of body condition score and cresty neck scoring system, (*iv*) palpation and visual assessment of the hoof capsule, (*v*) X‐ray examination, (*vi*) resting insulin levels, (*vii*) combined glucose‐insulin test, and (*viii*) leptin concentration as previously described by Basinska *et al*. 6.

**Table 1:** Criteria for dividing the horses into the experimental and control group

Group	O (number)	Sex	Main clinical parameters					
			Bw (kg)	BCS (1–9)	CNS (1–5)	Fasting insulin (mU/ml)	LEP (ng/ml)	CGIT:GLU in 45 min. (mg/dl)
Healthy horse	1	f	610	6	1	7	3.21	74/p
	2	f	644	7	2	12	4.12	69/p
	3	f	627	7	2	9	2.87	71/p
	4	m	609	6	1	8	1.86	89/p
	5	m	649	7	2	14	3.56	80/p
	6	m	639	6	2	13	2.91	74/p
Mean ± S.D.			629.7 ± 15.7	6.5 ± 0.5	1.7 ± 0.5	10.5 ± 2.6	3.1 ± 0.7	76.2 ± 6.7
Horse with EMS	1	f	710	8	3	83	4.89	138/p
	2	f	726	9	3	67	5.19	141/p
	3	f	760	9	4	98	9.12	140/p
	4	m	709	8	3	73	8.49	136/p
	5	m	716	8	4	69	7.27	134/p
	6	m	746	9	4	82	8.36	146/p
Mean ± S.D.			727.8 ± 19.1	8.5 ± 0.5	3.5 ± 0.5	78.7 ± 10.5	7.2 ± 1.6	139.2 ± 3.8

f: female; m: male; BW: bodyweight; BCS: body condition score; CNS: cresty neck score; CGIT: combined glucose‐insulin test; S.D.: standard deviation; LEP: leptin; GLU: glucose; p: positive test results; n: negative test results. As presented before by Basinska *et al*. 6 and Marycz *et al*. 34.

### Isolation of equine ASCs

White, subcutaneous adipose tissue (2 g) was collected from the horses' tail base, according to the standard surgical procedure and ethical standards, as previously described 6. After harvesting, specimens were placed in a sterile Hank's balanced salt solution (HBSS).

Isolation of ASCs was performed in accordance to a previously described protocol 16. Briefly, tissue fragments were washed extensively with HBSS supplemented with 1% antibiotic‐antimycotic solution (penicillin/streptomycin/amphotericin B; P/S/A) and minced. The minced tissue was digested in collagenase type I solution (1 mg/ml) for 40 min. at 37°C. After centrifugation (1200 × g, 10 min.) the supernatant was discarded and the pellet containing the cells was re‐suspended in culture medium and placed in a cell culture flask. Primary culture of ASCs' was designated as ‘passage 0’. To prepare cells for experiment, they were passaged three times.

### Cell culture

Cells were maintained at constant conditions in an incubator (37°C, 5% CO_2_) throughout the experiment. The primary cultures were plated on T‐25 culture flasks and cultured in DMEM with the F‐12 Ham nutrient, 10% foetal bovine serum (FBS) and 1% PSA solution. DMEM containing 4500 mg/l glucose supplemented with 10% FBS and 1% of PSA was used in the secondary cultures. The media were changed every 2 days and adherent cells were detached from the flask using TrypLETM Express (Life Technologies, Warsaw, Poland) after reaching 80% confluence.

### Immunophenotyping and multipotency assay

Isolated cells were characterized by checking the expression of the following surface markers: CD44, CD45, CD90 and CD105. The purity of ASCs was verified using Fluorescent‐Activated cell sorting (BD FACSCalibur, Becton Dickinson, Franklin Lakes, New Jersey, USA). Due to immunophenotyping ASCs were detached using TrypLETM Express solution, washed with HBSS contained 2% FBS and re‐suspended at total of 5*105 cells/ml. Cell suspension was incubated at 4°C for 20 min. with the specific primary antibodies at the dilution of 1:500 (anti‐CD105, Acris, Herford, Germany, SM1177PT; anti‐CD45, Novus Biologicals, Littleton, Colorado, USA, NB1006590APC, anti‐CD44, R&D Systems, Minneapolis, Minnesota, USA, MAB5449, anti‐CD90, ab225; Abcam, Cambridge, UK). Next, the cells were washed three times by centrifugation at 400 × g for 5 min. and re‐suspend in fluorochrome‐labelled secondary antibody (Alexa Fluor 488, ab150113; Abcam). At least ten thousand stained cells were acquired and analysed by FACS Calibur flow cytometer. The samples were analysed using CellQuest Pro software (Becton Dickinson, Franklin Lanes, New Jersey, USA).

Multipotency of isolated ASCs was confirmed by osteogenic, chondrogenic and adipogenic differentiation of cells cultured in StemXVivo kits (R&D System). In order to perform the assay, cells were maintained in 24‐well plates and inoculated at concentration of 1 × 10^4^ cells per well. The media was changed every 2 days. Chondrogenic and osteogenic stimulation was conducted for 11 days, whereas cells were cultured in adipogenic medium for 9 days. Cultures expanded in the standard growth medium were used as a control to establish the effectiveness of the differentiation process.

Prior to staining, the cells were fixed with 4% paraformaldehyde (PFA). In order to identify the formation of intracellular lipid droplets, cells were stained with LipidTOX dye (Thermo Fisher Scientific, Waltham, Massachusetts, USA). After chondrogenic differentiation, cells were stained with 0.1% aqueous solution of Safranin O (specific for proteoglycans). To visualize extracellular matrix mineralization formed in the course of osteogenic differentiation, the cells were stained with Alizarin Red. Cells were observed under an inverted microscope (AxioObserverA1; Zeiss, Oberkochen, Germany) and pictures were taken using Cannon PowerShot digital camera.

Detailed cell morphology was evaluated using SEM (EVO LS15; Zeiss). In order to perform observations cells were rinsed with distilled water and dehydrated in graded ethanol series (concentrations from 50% to 100%). Dehydrated samples were then sputtered with gold (Scancoat Six, Crawley, United Kingdom), placed in a microscope chamber and observed using the SE1 detector, at 10 kV of filament's tension.

### Cell viability, population doubling time and colony forming unit‐fibroblasts assay

The proliferation rate of ASCs was evaluated using a resazurin assay kit (TOX8), following the manufacturers protocol. To perform the test, cells were plated in 24‐well plates at a concentration of 2*10^4^ per well and inoculated with 0.5‐ml volume of culture medium. Next, culture media were collected and replaced with medium containing 10% of resazurin dye. Then cultures were incubated with a dye in a CO_2_ incubator, 37°C for 2 hrs. Supernatants were collected and transferred to 96‐well plate to perform the spectrophotometric assay (BMG Labtech, Ortenberg, Germany). The absorbance of the supernatants was measured at a wavelength of 600 nm for resazurin, and 690 nm reference wavelength. Each measurement included a blank sample, containing medium without cells. The number of cells was estimated on the basis of standard curve, generated during the experiment. To prepare the curve, cells were seeded at the density of 20 × 10^3^, 40 × 10^3^, 80 × 10^3^ per well and dye absorbance was measured in relation to certain cells number. Linear trendline equation allowed estimating cells number throughout the experiment. Number of cells cultured in standard condition was evaluated after 1, 4 and 7 days of the experiment, while after 1, 4, 7 and 11 days in osteogenic culture. Additionally, population doubling time (PDT) was assessed with the support of a population doubling time on‐line calculator 43 using formula presented below. Initial concentration equalled 2 × 10^4^ cells (initial seeding density) while final concentration equalled the number of cells in culture on the 7th day of the experiment.

$$PDT=\frac{\text{duration}*\log(2)}{\log(\text{Final Concentration})-\log(\text{Initial Concentration})}$$

To evaluate the ability to form colonies, the colony forming unit‐fibroblastic assay (CFU‐F) was performed as described previously with modifications 44. Briefly, the cells were seeded in 6‐well plates at a density of 10^2^ per well, inoculated into a culture medium and propagated for 7 days. After fixation in 4% ice‐cold paraformaldehyde, cells were stained with pararosaniline and colonies of more than 50 cells were scored. The efficiency of colony forming (CFE) ability was calculated using the formula mentioned below, as presented elsewhere 45.

$$CFUFs\left[\%\right]=\left(\frac{\text{number of colonies}}{\text{initial cell number}}\right)\cdot 100\%$$

### Evaluation of cells morphology, ultrastructure and morphomethry

Cells' morphology (induced and non‐induced) was evaluated using an epi‐fluorescent microscope (Axio Observer A.1; Zeiss), scanning electron microscope (SEM, Zeiss Evo LS 15), transmission electron microscopy (FE‐STEM Auriga60) and confocal microscopy (Observer Z1 Confocal Spinning Disc V.2 Zeiss with live imaging chamber). Morphology analysis was performed on the 7th (non‐induced cells), and 11th day (cells from osteogenic differentiation). Prior to fluorescent microscopy observations samples were first (*i*) rinsed three times with HBSS; (*ii*) fixed with 4% paraformaldehyde for 45 min.; (*iii*) washed again (as described above) and (*iv*) permeabilized for 15 min. with 0.1% Triton X‐100 at room temperature and washed again. Actin filaments were stained using atto‐488‐labelled phalloidin at dilution 1:800 with HBSS for 40 min. in the dark at room temperature and cells' nuclei were counterstained with 4′,6‐diamidino‐2‐phenylindole (DAPI; 1:1000; Sigma‐Aldrich) for 5 min.

Detailed characteristics of ASCs morphology were evaluated using scanning electron microscope (Zeiss EVO LS15). After fixation in 4% PFA cells were washed with HBSS three times and dehydrated in a graded ethanol series (from 50% to 100%). Air‐dried samples were then sputtered with gold (ScanCoat 6; Oxford), placed in a microscope chamber and observed using a SE1 detector, at 10 kV of filament's tension.

To perform TEM analysis, the samples were collected and fixed with 2.5% glutaraldehyde for 24 hrs at 4°C. Then the cells were washed three times with distilled water and incubated for 2 hrs with 1% osmium tetroxide. The samples were then counterstained with lead citrate and uranyl acetate, dehydrated in a graded series of acetone, and embedded using an Agar Low Viscosity Resin Kit (Agar Scientific Ltd, Essex, UK). The specimens were incubated 48 hrs at 60°C to polymerize and subsequently were sectioned into ultrathin slices (70 nm), followed by collecting on copper grids. The observations were carried out using FE‐STEM Auriga60 at 20 kV filament tension. Evaluations of mitochondrial morphology and autophagosomes formation were accomplished by taking TEM micrographs from randomly selected areas of ASC. Additionally percentage of abnormal mitochondria (membrane raptures, vacuoles formation) in cells was calculated.

The diameter of cell nuclei observed under SEM was calculated based on the representative images. We decided to call the cell nuclei ‘enlarged’ when its diameter was greater than 21 μm as mean ASCs (isolated from healthy individuals) nuclei diameter equal to 16.4 μm as previously described by Grzesiak *et al*. 16 while ‘enlarged cells’ had diameter greater than 60 μm. Moreover, using SEM/EDX technique, we estimated number and size of bone nodules in each age group.

### Oxidative stress factors and adenosine triphosphate levels analysis

To evaluate stress levels, cells were cultured in normal growth media without phenol red for 2 days. Nitric oxide concentration was assessed using commercially available Griess reagent kit (Life Technologies). Superoxide dismutase activity was measured using a SOD Assay kit (Sigma‐Aldrich). Reactive oxygen species were estimated by incubating cells with an H2DCF‐DA (Life Technologies). The amount of adenosine triphosphate (ATP) produced by investigated cells was performed using ATP assay kit (Abcam). All procedures were performed according to manufacturer's protocols.

### Autophagy assessment

Using RT‐PCR we evaluated the expression of autophagy‐related genes including LAMP2, Beclin, LC3, Parkin. Additionally LAMP2 amount in investigated cells was evaluated using flow cytometer. Briefly, cells were detached from culture plate and centrifuged (350 × g for 5 min.) followed by the 10 min. fixation in 4% ice cold PFA. After washing, cells were then incubated in 0.1% Tween‐20 in HBSS for 20 min. After another wash, cells were incubated with anti LAMP2 antibody (ab25631; Abcam) diluted 1:200 in HBSS containing 10% Goat Serum for 30 min. at 22°C. Next cells were washed again and incubated with Alexa 488 goat anti‐mouse secondary antibodies (1:500, Alexa Fluor 488, ab150113; Abcam) for 30 min. at 22°C. At least five thousand stained cells were acquired and analysed by FACS Calibur flow cytometer. The samples were analysed using CellQuest Pro software. Moreover, using the same anti‐LAMP2 antibody, immunofluorescence staining of investigated cells was performed. Additionally, mitochondria were counterstained with MitoRed to investigate mitophagy. Cell's nuclei were stained with DAPI. Cells were observed and photographed using confocal microscopy (Observer Z1 Confocal Spinning Disc V.2 Zeiss with live imaging chamber). The fluorescence intensity was calculated using ImageJ software. Using TEM imaging we also visualized autophagosomes formation (FE‐STEM Auriga60).

### Immunofluorescence staining's

For visualization of both, mitochondria and ROS, ASCs were cultured in medium containing MitoRed dye (1:1000) and Total ROS detection kit for microscopy and flow cytometry (Enzo Life Sciences New York, New York, USA). for 30 min. at 37°C. Following washes, cells were fixed with 4% paraformaldehyde for 20 min. and then treated with 0.5% Triton X‐100 for 20 min. Following HBSS wash, unspecific binding sites were blocked with blocking buffer (10% Goat Serum, 0.2% Tween‐20 in HBSS) for 45 min. Following blocking cells were incubated with specific primary anti‐cytochrome c antibodies diluted 1:1000 in HBSS containing 1% Goat Serum and 0.2% Tween‐20 overnight (NB100‐56503; Novus Biologicals, Littleton, Colorado, USA).

To visualize 5‐methylocytosine (5‐mC) and 5‐hydroxymethylocytosine (5‐hmC) cells were: (*i*) fixed with 4% PFA for 40 min., (*ii*) permeabilized in 0.5% Triton X‐100 in HBSS, (*iii*) incubated with 4 N HCl for 15 min., and (*iv*) incubated with blocking buffer (10% Goat Serum, 0.2% Tween‐20 in HBSS) for 45 min. Next, cells were incubated with primary antibodies diluted 1:200 in HBSS containing 1% Goat Serum and 0.2% Tween‐20 overnight (anti‐5‐methylocytosine, ab73938; Abcam; anti‐5‐hydroxymethylocytosine, ab106918; Abcam).

Alexa 594 goat anti‐mouse and/or Alexa 488 goat anti‐rat, Alexa 488 goat anti‐mouse secondary antibodies were then applied for 1 hr at room temperature as appropriate (1:1000, goat anti‐rat, ab150157, goat anti‐mouse, ab150113, goat anti‐mouse, ab150116; Abcam).

Cell nuclei were counterstained with DAPI.

Moreover, we performed *in situ* BrdU‐Red DNA Fragmentation (TUNEL) assay (Abcam) to evaluate the level of apoptosis in investigated cultures. All procedures were performed following manufacturer's protocols. Based on the representative images percentage of TUNEL positive cells was calculated.

### Assessment of ASC secretory activity‐ELISA p53, VEGF, IL‐1α, IL‐1β and ALP activity

To evaluate the extracellular levels of secreted proteins, ELISA was performed. In order to evaluate the amount of Tumour protein p53 (p53), VEGF, IL‐1α and IL‐1β, supernatants were collected after 7th day from cells cultured in control medium. To evaluate the extracellular level of BMP‐2 and alkaline phosphatase (ALP), culture medium from 11th day of osteogenic differentiation was collected. All ELISA test were purchased from MyBioSource (San Diego, California, USA) and performed in accordance to manufacturer's instructions.

To estimate extracellular ALP activity, an Alkaline Phosphatase Colorimetric Assay Kit (Abcam) was used in according to manufacturer's protocol. Briefly, in the assay, the p‐nitrophenyl phosphate was used as a phosphatase substrate. The substrate was hydrolysed into p‐nitrophenol by ALP. The product was then measured spectroscopically at 405 nm wavelength (BMG Labtech). The amount of pNP was obtained by sample readings applied to a standard curve. ALP activity was calculated using the following formula: ALP activity (U/ml) = A/V/T (where A ‐ pNP amount; V ‐ volume of sample added to well (ml); T ‐ reaction time).

### Quantitative real‐time reverse transcription polymerase chain reaction

After 7th day of culture (viability test) and 11th day (osteogenic differentiation), the cells were rinsed with HBSS and homogenized by TriReagent^®^. Total RNA was isolated using phenol–chloroform method as previously described by Chomczynski and Sacchi 46. The obtained RNA was then diluted in DEPC‐treated water and analysed in terms of amount and quality using a nano‐spectrometer (WPA Biowave II). Preparation of DNA‐free RNA was performed using DNase I RNase‐free Kit (Thermo Scientific). For each reaction, 150 ng of total RNA was used. Complementary DNA (cDNA) was synthesized using a reverse transcriptase Tetro cDNA Synthesis Kit (Bioline, Cincinnati, Ohio, USA). Enzymatic digestion of total RNA and cDNA synthesis were performed in accordance with the manufacturers’ instructions using a T100 Thermal Cycler (Bio‐Rad, Hercules, California, USA).

The qRT‐PCR reactions were performed using a CFX ConnectTM Real‐Time PCR Detection System (Bio‐Rad). Reaction mixture contained 2 μl of cDNA in a total volume of 20 μl using SensiFast SYBR & Fluorescein Kit (Bioline). Primer concentration in each reaction equalled to 500 nM; primer sequences used in individual reactions are listed in Table 2. Relative gene expression analysis (Qn) was calculated in relation to the GAPDH housekeeping gene. Moreover, we evaluated the ratio between BCL‐2 and BAX expression in each group by dividing Qn of BCL‐2 by Qn of BAX. The ratio between LC‐3 and Beclin 1 expression in each group by dividing Qn of LC‐3 by Qn of Beclin 1.

**Table 2:** Sequences of primers used in qPCR

Gene	Primer	Sequence 5′–3′	Amplicon length (bp)	Accesion no.
Oct‐4	F:	TCTCTTTGGGAAGGTGTTCAG	190	XM_001490108.5
	R:	GTCTCAATACTAGTTCGCTTTCTC		
Parkin	F:	TCCCAGTGGAGGTCGATTCT	218	XM_005608126.2
	R:	CCCTCCAGGTGTGTTCGTTT		
PGC1 α	F:	TCTACCTAGGATGCATGG	93	XM_005608845.2
	R:	GTGCAAGTAGAAACACTGC		
HIF‐1‐α	F:	CTCAAATGCAAGAACCTGCTC	86	XM_014735822.1
	R:	TTCCATACCATCTTTTGTCACTG		
Bcl‐2	F:	TTCTTTGAGTTCGGTGGGGT	164	XM_001490436.3
	R:	GGGCCGTACAGTTCCACAA		
FOXO1	F:	ATTGAGCGCTTGGACTGTGA	311	XM_014732057.1
	R:	CGCTGCCAAGTTTGACGAAA		
LC3	F:	TTACTGCTTTGCTCTGCCAC	213	XM_005608485.2
	R:	AGCTGCTTCTCCCCCTTGT		
Beclin	F:	GATGCGTTATGCCCAGATGC	147	XM_014729146.1
	R:	ATCCAGCGAACACTCTTGGG		
LAMP2	F:	GCACCCCTGGGAAGTTCTTA	139	XM_014733098.1
	R:	TTCGAGGATCTGTGCCAATCA		
BAX	F:	TTCCGACGGCAACTTCAACT	218	XM_005596728.1
	R:	GGTGACCCAAAGTCGGAGAG		
p21	F:	GAAGAGAAACCCCCAGCTCC	241	XM_003365840.2
	R:	TGACTGCATCAAACCCCACA		
Coll‐1	F:	GAAACTATCAATGGTGGTACCAAGT	265	XM_008524258.1
	R:	AGCAGCCATCTACAAGAACAGT		
BMP‐2	F:	CGTCCTGAGCGAGTTCGAGT	249	XM_001493895.4
	R:	CGCCGGGTTGTTTTTCCACT		
Osteocalcin	F:	CGCTACCTGGATCATTGGCT	311	XM_014732057.1
	R:	GAGCAGCTAGGGACGATGAG		
RUNX‐2	F:	CCAAGTGGCAAGGTTCAACG	165	XM_005603968.1
	R:	TGTCTGTGCCTTCTGGGTTC		
PDK4	F:	GCTGGTTTTGGTTATGGCTTGC	137	XM_014853326.1
	R:	TCCACAGACTCAGAAGACAAAGCC		
Nanong	F:	CCTTAGCTACAAACAGGTTAAGAC	147	XM_001498808.1
	R:	TGGTGGTAGGAATAGAGCCC		
Sox‐2	F	GTTGTCAAGGCAGAGAAGAG	227	XM_015131530.1
	R:	GAGAGAGGCAAACTGGAATC		
p62	F:	CATCGGAGGATCCCAGTGTG	207	XM_005599173.2
	R:	CCGGTTTGTTAGGGTCGGAA		
GAPDH	F:	GATGCCCCAATGTTTGTGA	250	NM_001163856.1
	R:	AAGCAGGGATGATGTTCTGG		

Sequences, amplicon length and accession numbers of the primer sets. Oct‐4: octamer‐binding transcription factor 4; Parkin: parkin RBR E3 ubiquitin protein ligase (PARK2); PGC1 α: peroxisome proliferator‐activated receptor gamma, coactivator 1 alpha; HIF‐1‐α: hypoxia inducible factor 1, alpha subunit; Bcl‐2: B‐cell CLL/lymphoma 2; FOXO1: forkhead box O1; LC3: microtubule associated protein 1 light chain 3 beta (MAP1LC3B); Beclin: beclin 1, autophagy related (BECN1); LAMP2: lysosomal‐associated membrane protein 2; BAX: Bcl‐2 associated X protein; p21: Cyclin‐Dependent Kinase Inhibitor 1A; Coll‐1: collagen type I; BMP‐2: bone morphogenetic protein‐2; Osteocalcin: bone gamma‐carboxyglutamic acid‐containing protein, RUNX‐2: Runt‐Related Transcription Factor 2; PDK4: Pyruvate Dehydrogenase Kinase Isozyme 4; Nanog: Homebox Protein Nanog; Sox‐2: sex determining region Y‐box 2; p62: Sequestosome‐1; GADPH: Glyceraldehyde‐3‐Phosphate Dehydrogenase.

### Statistical analysis

Results are shown as growth curves or box plots of at least three independent experiments, measured as triplicates or more. Statistical significance was determined using the un‐paired *t*‐test or one‐way anova with Tukey's post hoc test (Prism5.04; GraphPad Software, La Jolla, California, USA). *P* < 0.05 was considered statistically significant.

## Results

### Immunophenotyping and multipotency assay

**Figure 1:**
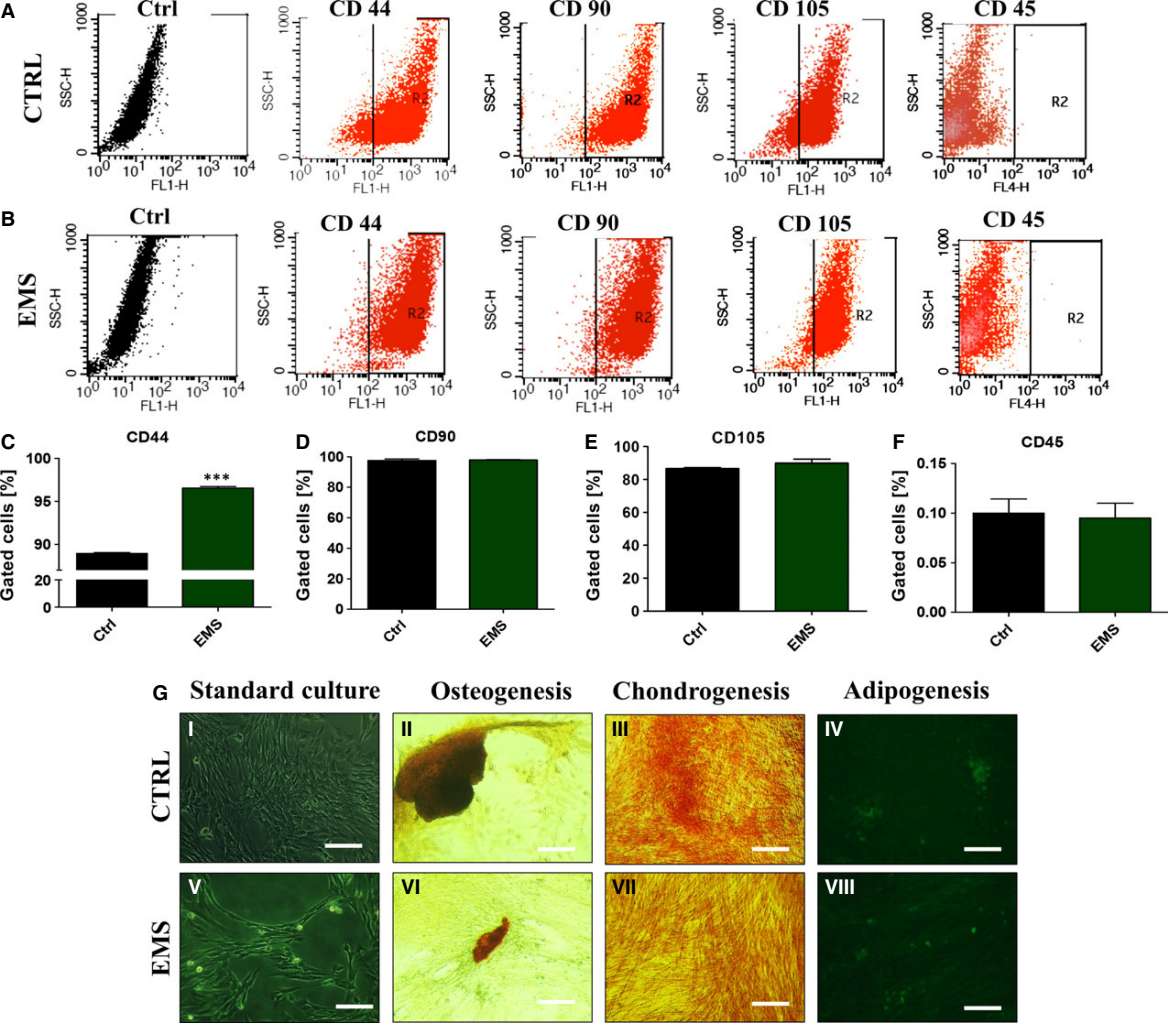
Flow cytometry and multipotency analysis. Flow cytometry dot plots representative for equine adipose derived mesenchymal stem cells (ASCs) from control (**A**) and EMS (**B**) group. Isolated cells are characterized by the presence of CD44, CD90, CD105 and CD45. ASCs showed the lack of expression CD45 surface markers (**A**,** B**,** F**). Flow cytometry analysis determined the percentage of specific markers in total analysed ASCs. Quantification of markers revealed significantly higher expression of CD44 in EMS group (*P* < 0.001, **C**). We did not observe differences in the expression of CD90 (**D**), CD105 (**E**) and CD45 (**F**), respectively. Representative images from tri‐lineage assay of ASCs differentiation (**G**). Cells cultured in control medium served as a control to monitor effectiveness of differentiation process (I, V). Osteogenically differentiated cells in monolayer stained with Alizarin Red (II, VI), chondrogenically differentiated cells using Safranin O (III, VII) and adipogenically differentiated cells stained using LipidTOX (IV, VIII). Magnification ×100, scale bars: 250 μm. Results expressed as mean ± S.D., ***P < 0.001.

Isolated cells presented typical for ASC characteristics features including (*i*) plastic adherent growth, (*ii*) expression of CD44, CD90 and CD105 and (*iii*) the lack of expression CD45 surface antigen (Fig. 1A and B). Interestingly, among all investigated antigens (Fig. 1D–F), only CD44 expression was statistically significant, as we observed it increased expression in ASC_EMS_ (Fig. 1C, *P* < 0.001).

Obtained ASCs differentiated into osteogenic, chondrogenic and adipogenic lineages, what was verified by means of specific staining (Fig. 1G). The formation of extracellular mineralized matrix was confirmed by Alizarin Red staining. Obtained photographs indicate on increased bone nodule size in control group. Similarly, the chondrogenic differentiation effectiveness was improved in control group as we observed increased proteoglycan accumulation stained with Safranin O in this group. Interestingly, LipidTOX staining revealed no differences in adipogenic lineage differentiation between investigated groups.

### Evaluation of ASCs morphology and the expression of Oct‐4, SOX‐2 and NANOG

**Figure 2:**
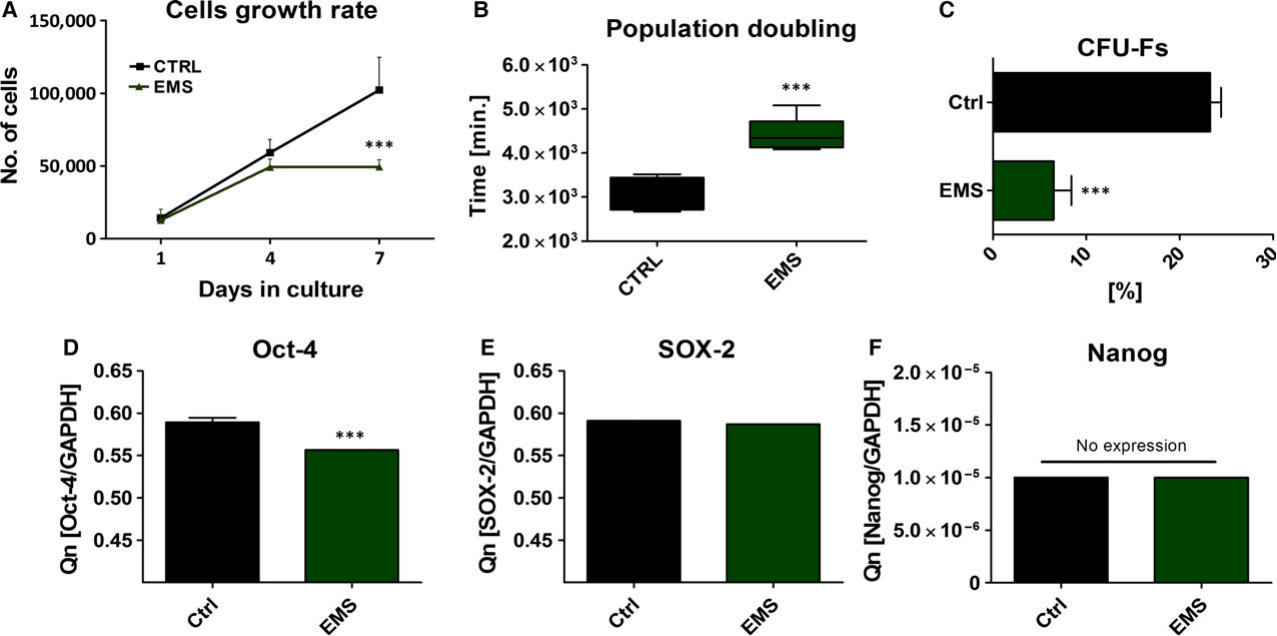
Evaluation of growth kinetics, PDT and clonogenic potential of ASCs. Mean cell number for each group with respect to culture duration (**A**). ASC_EMS_ showed decreased proliferation potential, especially on seventh day of culture (*P* < 0.001). In respect to growth curve data, mean population doubling time was calculated (**B**). CFU‐E assay showing percentage of colonies consisting of more than 50 cells between groups after 7 days of culture (**C**). The expression of Oct‐4 (**D**), Sox‐2 (**E**) and Nanog (**F**) evaluated with RT‐PCR after day 7th. Obtained data indicate on decreased proliferation rate, clonogenic potential and ‘stemness’ of ASC_EMS_. Results are expressed as mean ± S.D. ***P < 0.001.

Viability characteristics of ASCs were assessed within 7 days of culture. The number of viable cells in culture was evaluated with resazurin‐based assay (TOX‐8) in accordance to the manufacturer's protocol. The initial cell number, on the first day of experiment amounted to 1 × 10^3^ cells/well. Throughout the experiment, ASC_EMS_, displayed lowest proliferation rate in comparison to control group, but only on day 7th the difference between groups was statistically significant (Fig. 2A, *P* < 0.001). Time required to double the population‐ PDT‐ was greatest in this group in comparison to control (Fig. 2B, *P* < 0.001). The ability of cells to form colonies originated from one cell, was established with clonogenic fibroblast precursor (CFU‐F) assay. Moreover, the number of colonies consisted of more than 50 cells was decreased in this group (Fig. 2C, *P* < 0.001). Additionally, RT‐PCR analysis revealed decreased expression of pluripotency‐related Oct‐4 in ASC_EMS_ (Fig. 2D, *P* < 0.01) and no differences in Sox‐2 expression. Moreover, we performed RT‐PCR analysis for NANOG, but no expression was observed (Fig. 2F).

**Figure 3:**
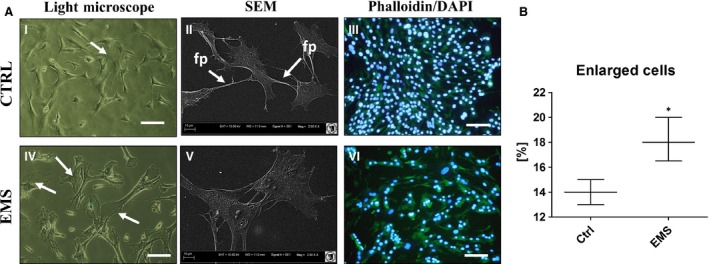
Assessment of ASCs morphology. Using multiple types of cells imaging allowed to visualize cells morphology in investigated groups (**A**) after 7 days of culture. Light microscope (I, IV) pictures with white arrows indicating cells with enlarged bodies as a sign of cellular senescence and apoptosis. SEM images (II, V) confirmed that ASC_EMS_ were characterized by more flattened and spread‐out shape. Moreover, in control group we observed long cytoskeletal projection called filopodia (indicated with white arrows as fp), which connect neighbouring cells and plays crucial role in intracellular signalling. Staining for actin filaments and nuclei (III, VI) revealed that in control group cells were concentrated more densely and closely adhere to each other and formed multi‐layer, whereas in EMS group cells reached about 70% of confluence. Moreover, some of those cells were amorphous in shape and no longer bipolar with enlarged nuclei. Additionally, based on representative photographs, we calculated percentage of enlarged cells, which was elevated in EMS group (**B**). Magnification ×100 (light and fluorescence microscopy), scale bars: 250 μm. Results expressed as mean ± S.D., *P < 0.05.

Cell morphology was evaluated on the first and last (7th) day of the experiment (Fig. 3A). Observations with light microscope showed that, ASC_CTRL_ displayed more fibroblast‐like, elongated morphology, whereas ASC_EMS_ were rather flat and no longer of bipolar shape (Fig. 3A I, IV). SEM analysis revealed that, ASC_CTRL_ developed greatest net of cytoskeletal projections — long structures known as lamellipodia and filopodia, which connected adjacent cells, in comparison to ASC_EMS_ (Fig. 3A II, V). Staining with DAPI and Phalloidin after 168 hrs revealed multi‐layer formation by ASC_CTRL_, whereas the colony confluence in ASC_EMS_ reached approximately 70% (Fig. 3A III, VI). Based on the representative photographs, we calculated percentage of cells with enlarged body, which stands for one of the signs of cellular senescence and apoptosis). The number of spread‐out, flattened cells was increased in ASC_EMS_ group (Fig. 3B, *P* < 0.05).

### Evaluation of ASCs secretory activity

**Figure 4:**
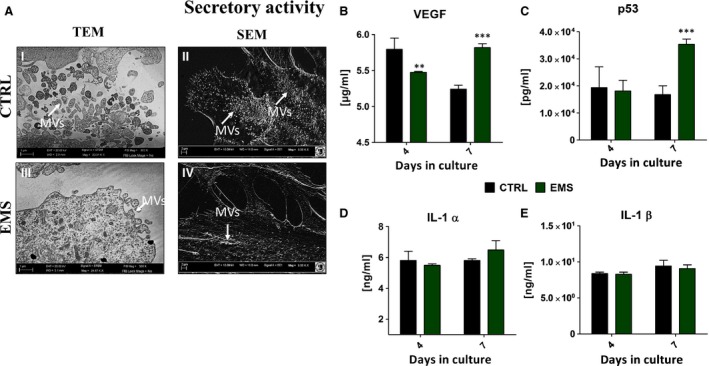
Secretory activity of ASCs. Transmission and electron microscopy images showing decreased production of MVs by ASC_EMS_ (**A**) after 7 days of propagation. White arrows pointing on MVs (MVs‐ membrane‐derived microvesicles). Additionally, using ELISA we established extracellular amount of VEGF (**B**), p53 (**C**), IL‐1α (**D**) and IL‐1β (**E**). Results expressed as mean ± S.D. **P < 0.01, ***P < 0.001.

One of the potential explanations of the regenerative ability of ASCs is paracrine action through secretion of MVs which contain a wide range of growth factors, as well as anti‐apoptotic and anti‐inflammatory factors. Thus, using transmission and electron microscopy we imaged the synthesis of MVs by investigated cells. The secretion of MVs was more robust in control groups, MVs were localized not only on the edge of cells body but also in the area of nucleus (Fig. 4A I, II). On the contrary, the number of MVs produced by ASC_EMS_ was reduced (Fig. 4A III, IV).

Moreover, we evaluated the levels of secreted form of following proteins: VEGF, p53, IL‐1α and IL‐1β. Interestingly, VEGF level was decreased in ASC_EMS_ after 4th day (Fig. 4B, *P* < 0.01) but it amount after 168 hrs was significantly higher than in control group (*P* < 0.001). This may indicate on lower metabolic activity of ASC_EMS_, as they need more time to synthesize proteins. Those group was also characterized by increased expression of p53 after 168 hrs (*P* < 0.001) in comparison to control group (Fig. 4C). Interestingly there were no significant differences in, IL‐1α and IL‐1β production among groups.

### Increased methylation status in ASC_EMS_

**Figure 5:**
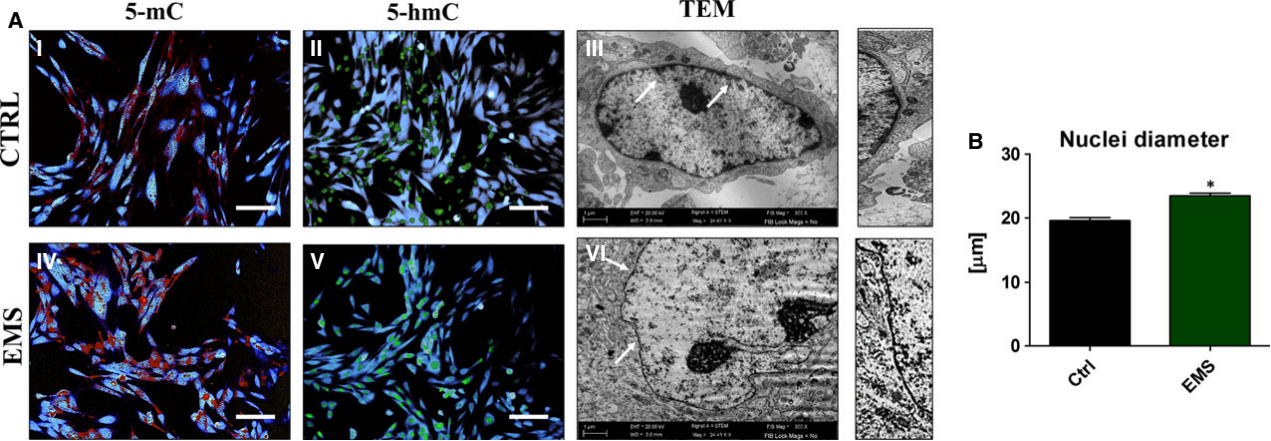
Genomic distribution of 5‐mC and 5‐hmC in ASC. Genomic distribution of 5‐MC (I, IV) and 5‐hMC (II, V) was evaluated after day 7th using immunofluorescence stainings in order to determine DNA methylation status. Nuclei were counterstained with DAPI. Obtained results indicates on increased methylation in ASC_EMS_. Additionally, using TEM we visualize the morphology of nuclei (III, VI). White arrows indicate on heterochromatin architecture at the nuclear periphery, thinner in ASC_EMS_ in comparison to control. Higher magnification images of indicated with arrows regions are shown on the right. Magnification ×100 (fluorescence microscopy), scale bars: 250 μm. Results expressed as mean ± S.D., *P < 0.05.

Genomic distribution of 5‐mC and 5‐hmC was evaluated by immunofluorescence staining after 7th day of culture to assess DNA methylation status. Nuclei were counterstained with DAPI (Fig. 5A). The 5‐mC fraction was more robust in ASC_EMS_ (Fig. 5A I) group with simultaneously decreased levels of 5‐hmC (Fig. 5A II) in comparison to control group (Fig. 5A III, IV). Interestingly, TEM images revealed that, ASC_EMS_ showed reduced heterochromatin architecture at the nuclear periphery, which may leads to mitotic defects comprising delays in cytokinesis and nuclear reassembly, abnormal chromosome segregation, and binucleation. Using TEM imaging, we estimated mean nuclei diameters in both ASC_CTRL_ and ASC_EMS_ (Fig. 5B). The nuclei diameters were increased in ASC_EMS_ (Fig. 5B).

### Oxidative stress analysis revealed increased ROS production and abnormal mitochondria in ASC_EMS_

**Figure 6:**
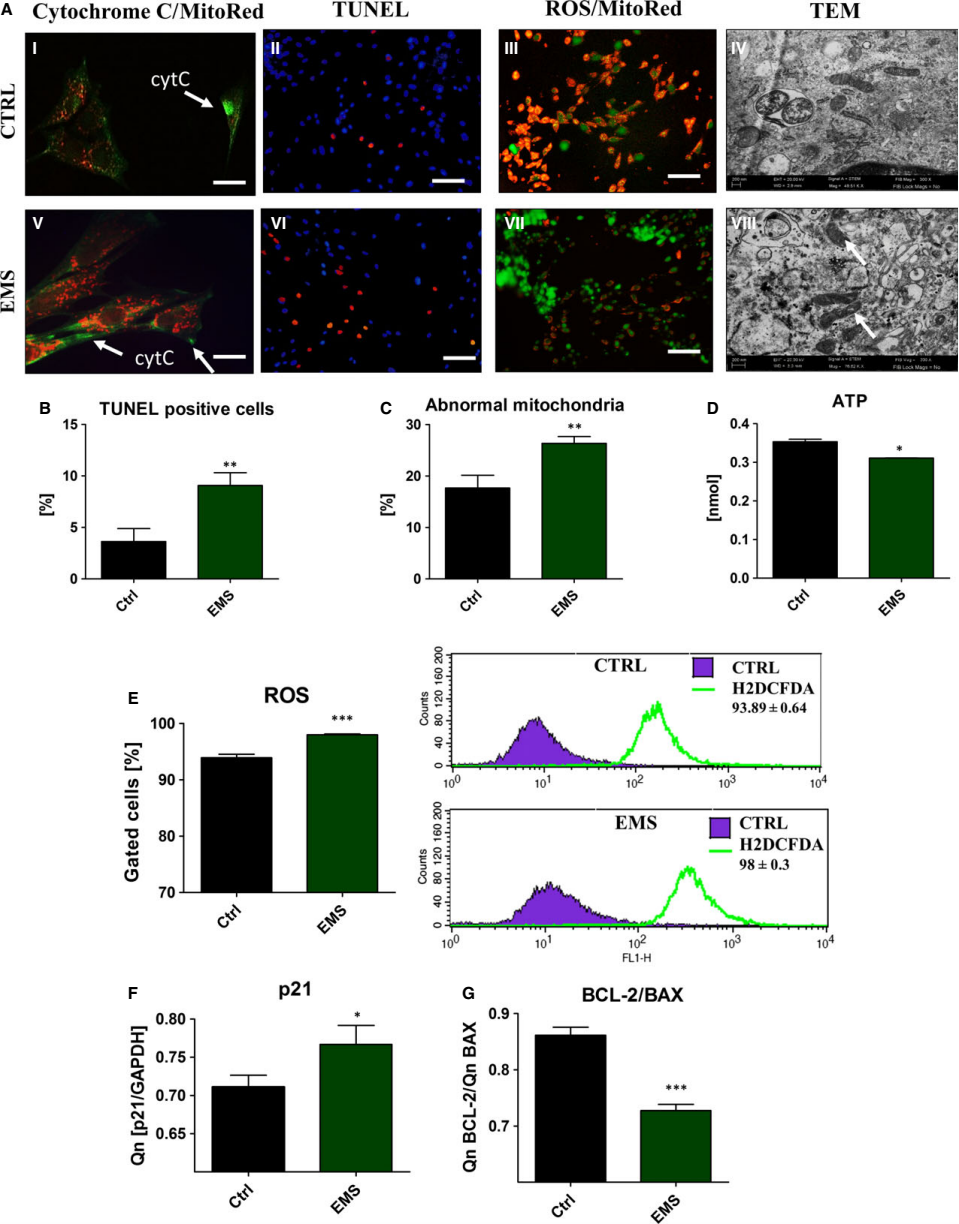
Oxidative stress and mitochondria morphology in ASC after 7 days of culture. ASC were stained with cytochrome c antibody and MitoRed dye to determine the subcellular localization of cytochrome c (green) and mitochondria (red), cytC‐ cytochrome c (A). Representative pictures shows cytochrome c relocation to cytoplasm in ASC_EMS_. To evaluate the levels of apoptosis in investigated cultures TUNEL staining was performed (II, VI). Merged pictures revealed that most of ROS was derived from mitochondria (III, VII). To determine if EMS affects mitochondrial structure in ASC, transmission electron microscopy was performed. Representative photographs (IV, VIII) showed accumulation of abnormal mitochondria in ASC_EMS_. White arrows indicate on mitochondria with disarrayed cristae, membrane raptures and vacuoles within the matrix. Those defects have been correlated with mitochondrial fission, metabolic disorders and cell death. Based on the representative photographs TUNEL positive cells were calculated (**B**). Percentage of abnormal mitochondria with membrane raptures and vacuole formation was calculated (**C**) based on TEM photographs. Moreover, we observed decreased ATP levels in ASC_EMS_, which may result from dysfunctional mitochondria in those cells (**D**). Additionally, to evaluate whether mitochondria dysfunction influence oxidative status of ASC and accumulation of intracellular ROS levels were evaluated with flow cytometer (**E**). Flow cytometer histograms are shown next to the box plot. Using RT‐PCR we also established expression of p21 (**F**), which was increased in ASC_EMS_ (*P* < 0.05). Moreover, the ratio of BCL‐2/BAX expression was significantly lower (*P* < 0.001) in ASC_EMS_ making those cells more susceptible for apoptosis (**G**). Magnification ×100 (fluorescence microscopy), scale bars: 250 μm. Results expressed as mean ± S.D., *P < 0.05, **P < 0.01, ***P < 0.001.

To evaluate if cells undergo intrinsic apoptotic pathway, we examined whether the subcellular localization of cytochrome c was altered in ASC_EMS_. Additionally, mitochondria were counterstained with MitoRed (Fig. 6A I, III, V, VII). Confocal microscopy photographs revealed apoptotic cells within ASC_EMS_ population with cytochrome c loss from the mitochondria and its translocation to cytoplasm (Fig. 6A I, V). Additionally, we observed increased number of TUNEL‐ positive cells in ASC_EMS_ (Fig. 6A II, VI, B) in comparison to control group (*P* < 0.01). Moreover, we performed staining for ROS and we observed that ROS levels were increased in ASC_EMS_. When comparing ROS images with Mito Red staining, it is apparent that the most ROS is derived from mitochondria. Based on TEM images percentage of abnormal mitochondria in cells was calculated (Fig. 6C). Moreover, we observed decreased production of ATP in ASC_EMS_ (*P* < 0.05; Fig. 6D). Additionally ROS levels was evaluated using flow cytometry (Fig. 6E) and obtained results confirmed increased formation of intracellular ROS in ASC_EMS_. Moreover using TEM, we observed mitochondria alternations within ASC_EMS_ cells. Those mitochondria were characterized by (*i*) smaller shape and disarrayed cristae, (*ii*) membrane raptures and (*iii*) vacuoles formation. The expression of p21, protein related to oxidative stress response, was increased in ASC_EMS_ (Fig. 6F). Moreover the ratio of BCL‐2/BAX expression was significantly lower (*P* < 0.001) in ASC_EMS_ (Fig. 6G, *P* < 0.05).

### Osteogenic differentiation of ASC

**Figure 7:**
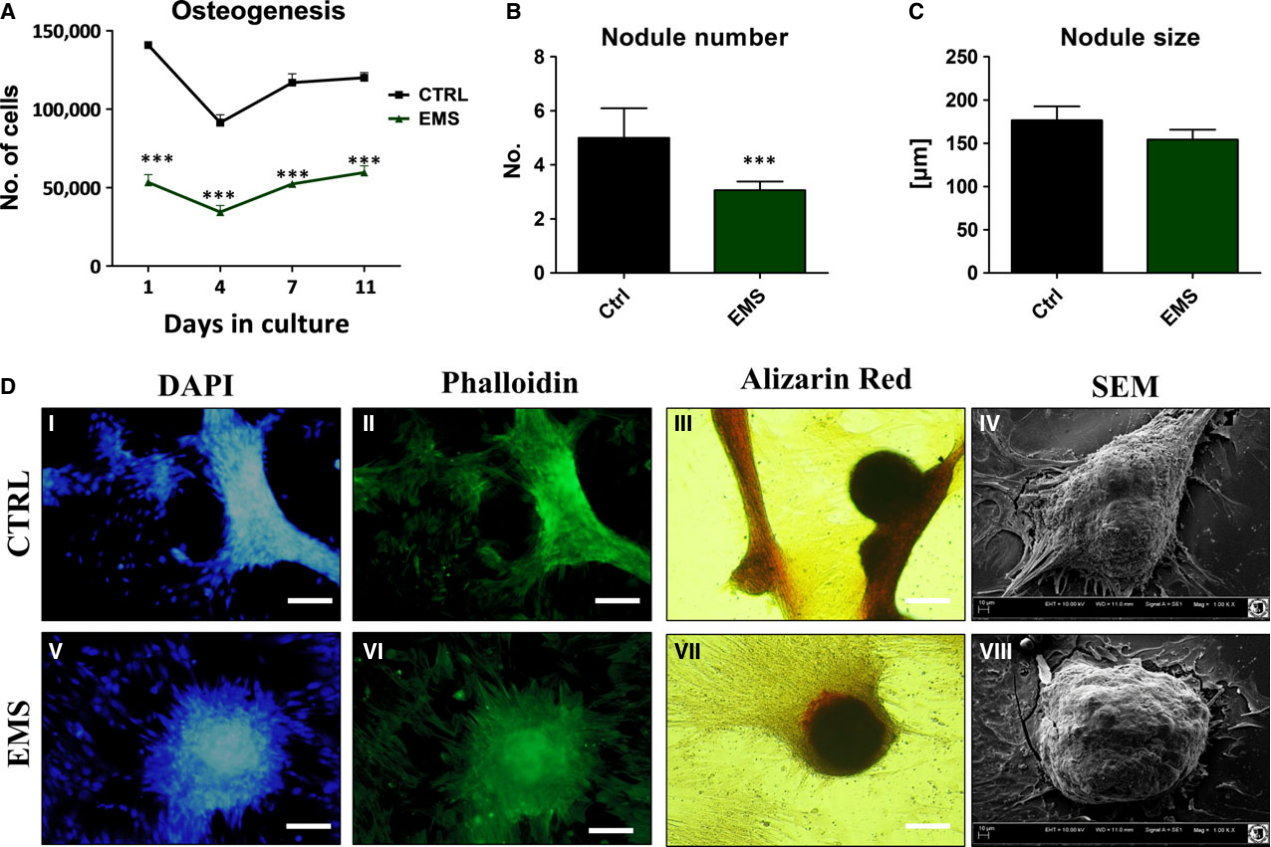
Osteogenic differentiation of ASC after 11 days of propagation. ASC_EMS_ displayed decreased proliferation rate during osteogenic culture in comparison to control group (**A**,* P* < 0.001). Based on the SEM results mean nodule number and size was estimated. Although, ASC_EMS_ produced less number of bone nodules (**B**,* P* < 0.001) we did not observed differences in their size among groups (**C**). Visualization of cellular morphology with DAPI/Phalloidin (I, II, V, VI) showed formation of dense cellular aggregates, whereas Alizarin Red stained extracellular mineralized matrix (III, VII). SEM pictures of bone nodules revealed no differences in their size among investigated cells (IV, VIII). Magnification ×100, scale bars: 250 μm. Results expressed as mean ± S.D. ***P < 0.001.

Adipose derived mesenchymal stem cells were cultured under osteogenic conditions in order to evaluate their osteogenic differentiation potential on a functional level (Fig. 7A). A significantly lower proliferative activity was observed in osteoblasts precursors (Op) derived from ASC_EMS_ throughout the whole experiment (*P* < 0.001). Additionally using SEM we evaluated the number and size of bone nodules within investigated cells. Hence, we observed that ASC_EMS_ were characterized by the formation of lowest number of bone nodules (Fig. 7B, *P* < 0.001) whereas there was no statistically important differences in nodule size among groups (Fig. 7C). Stainings with DAPI/Phalloidin and Alizarin Red confirmed formation of dense aggregates and mineralized matrix (Fig. 7D I, II, III, V, VI, VII). SEM imaging revealed that bone nodules in both groups presented similar size (Fig. 7D IV, VIII).

**Figure 8:**
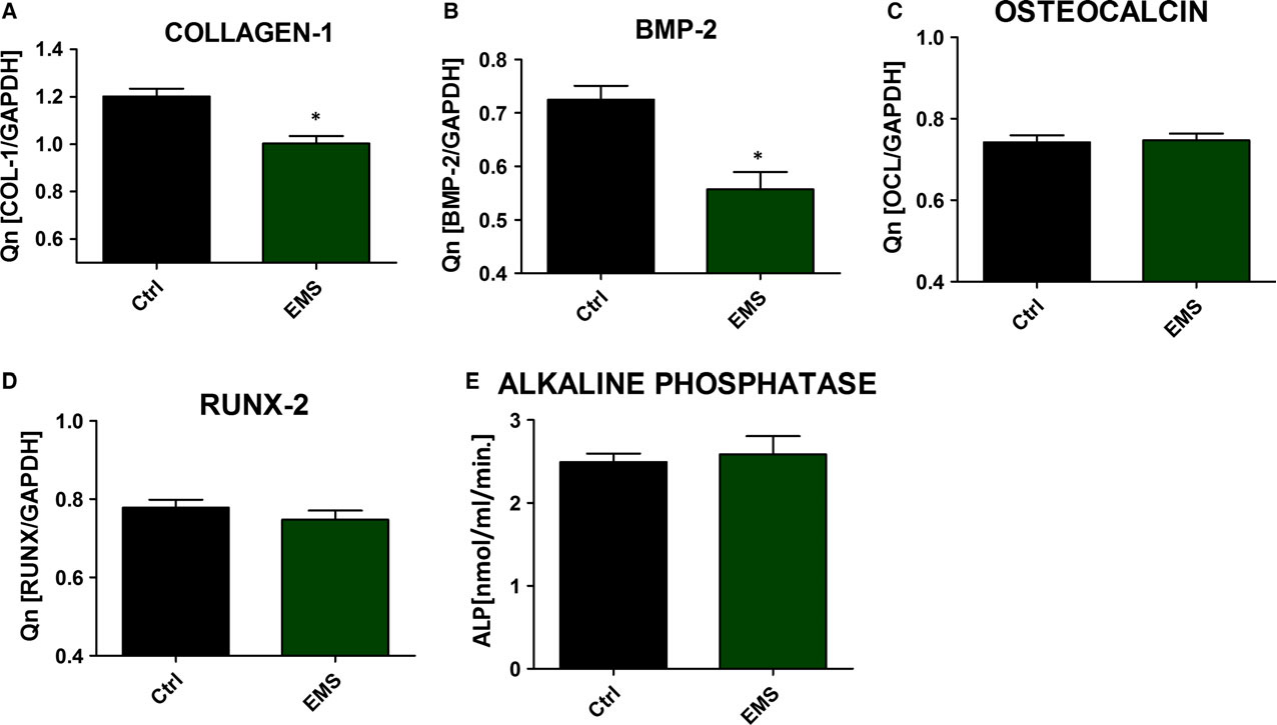
Evaluation of protein expression during osteogenic differentiation. RT‐PCR results for collagen‐1 (**A**), BMP‐2 (**B**), osteocalcin (**C**) and RUNX‐2 (**D**). Activity of alkaline phosphatase (**E**). Data were obtained from the 11th day of experiment. Results expressed as mean ± S.D., *P < 0.05.

Moreover, to establish effectiveness of differentiation process using RT‐PCR we evaluated the amount of collagen‐1, BMP‐2, osteocalcin and RUNX‐2 mRNA levels. Only collagen‐1 (Fig. 8A) and BMP‐2 (Fig. 8B) expression was importantly decreased in ASC_EMS_ (*P* < 0.05). No statistically important differences were observed in osteocalcin (Fig. 8C) and RUNX‐2 (Fig. 8D). Additionally, ALP amount was similar in both groups (Fig. 8E).

### Analysis of the oxidative stress factors in Op

**Figure 9:**
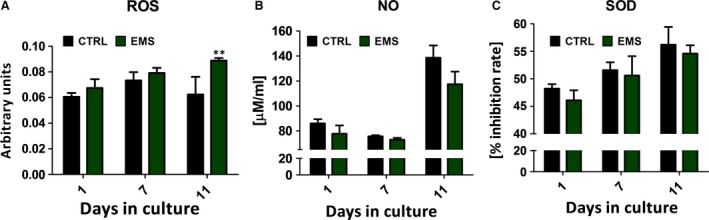
Analysis of the oxidative stress factors in osteoblast precursor (Op). Reactive oxygen species (**A**), nitric oxide (**B**), superoxide dismutase (**C**). Obtained results indicate on indicating on the existence of protective mechanism that switches on during this process in ASC_EMS_. Results expressed as mean ± S.D., **P < 0.01.

Oxidative stress factors *e.g*. ROS and nitric oxide and the activity of SOD was evaluated after 1st, 7th and 11th days of culture. Obtained results shows that during osteogenesis, the amount of ROS is similar between groups (Fig. 9A), and only on the last day of culture it was importantly elevated in ASC_EMS_ (*P* < 0.001). Interestingly, in the course of osteogenic differentiation process the amount of nitric oxide (Fig. 9B) and SOD (Fig. 9C) activity was comparable between groups indicating on the existence of protective mechanism enables during this process in ASC_EMS._

### Autophagy and mitochondrial biogenesis in control and osteogenic conditions

**Figure 10:**
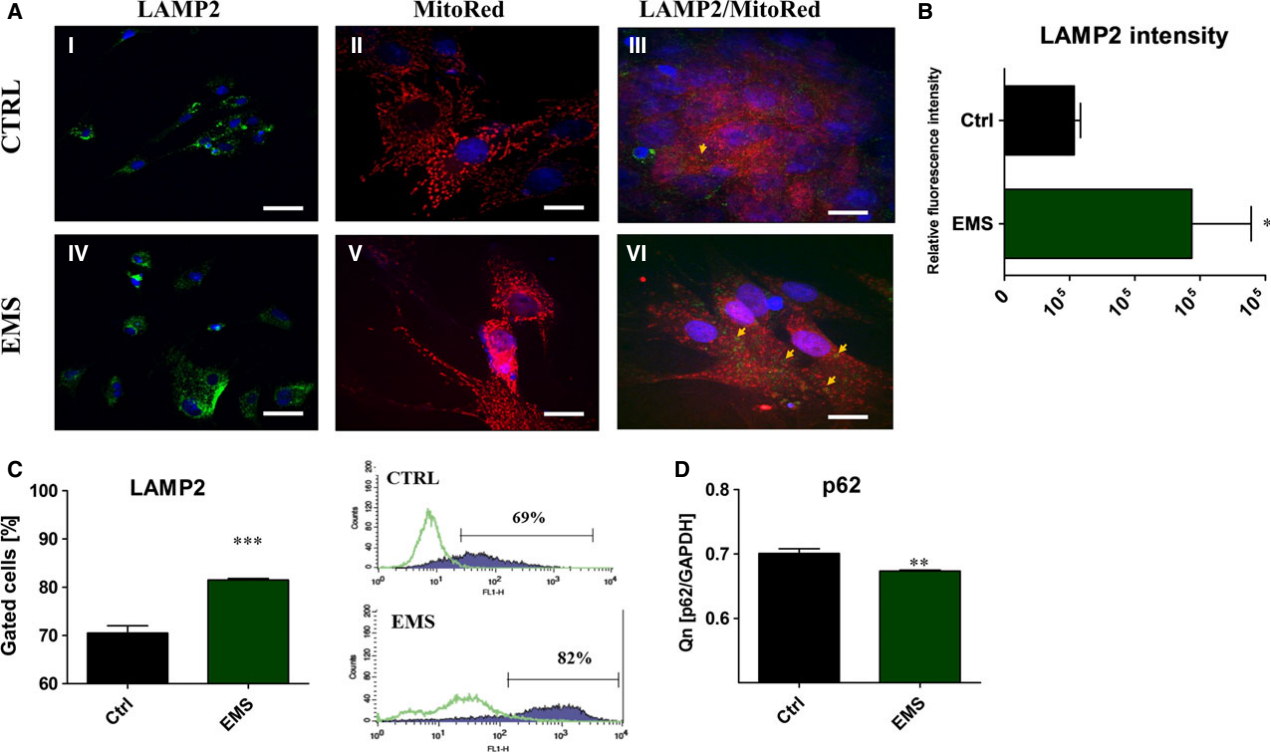
The evaluation of LAMP2 after 7 days of culture. Cells were stained with anti‐LAMP2 antibody and observed under confocal microscope (I, IV). Additionally mean fluorescence intensity of LAMP2 was evaluated (**B**). Moreover, using MitoRed we evaluated the net of mitochondria in investigated ASCs (II, V). In control group, mitochondria were more dense, robust, forming long, interconnected tubular network, located evenly within the cytoplasm. In ASC_EMS_ mitochondria were located mainly in the area of the nucleus and presented as a round, separate tubules. Moreover, merged photograph of MitoRed/LAMP2 revealed increased formation of mitophagosomes in ASC_EMS_ (III, VI). For more quantitative data LAPMP2 amount was estimated with flow cytometer (**C**). Obtained results confirmed immunofluorescence stainings. The amount of p62 was decreased in ASC_EMS_ (*P* < 0.01), indicating on increased autophagy in those cells (**D**). *P < 0.05, ***P < 0.001. Magnification ×10 LAMP2, scale bars 20 μm, MitoRed, Mitored/LAMP2 magnification ×20, scale bars 10 μm.

**Figure 11:**
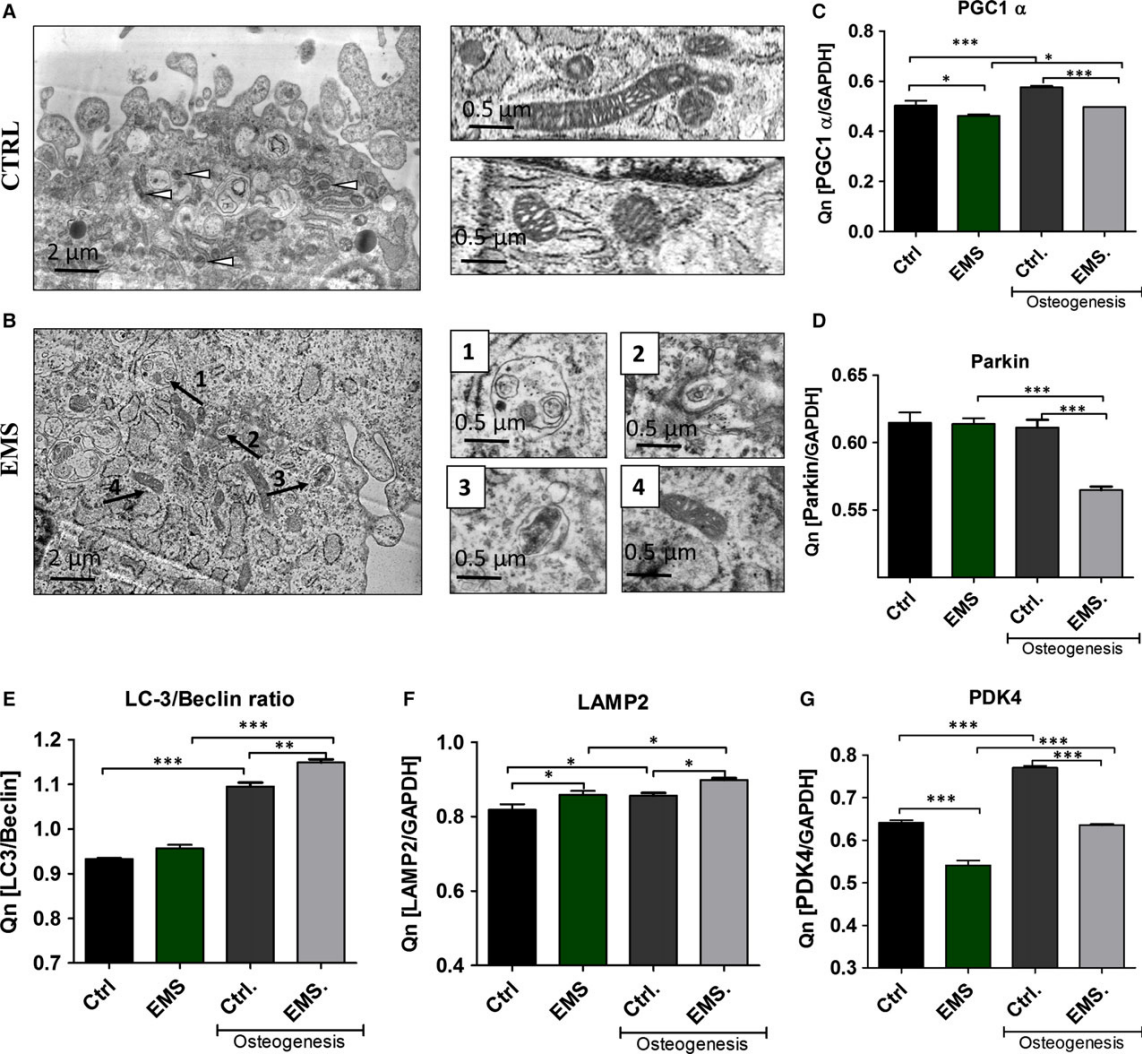
Autophagy and mitochondrial biogenesis in control and osteogenic conditions. TEM imaging revealed increased formation of autophagosomes and autolysosomes in ASC_EMS_ (**B**). Moreover, we observed clearance of dysfunctional mitochondria through autophagy. Higher magnification images of indicated with arrows regions are shown on the right. PGC1 α expression indicates on increased mitochondria biogenesis during osteogenesis in ASC (**C**), while Parkin expression on the removal of dysfunctional mitochondria by mitophagy (**D**). The ratio of LC‐3/Beclin 1 suggest increased autophagosome formation during osteogenesis (**E**) while LAMP‐2 expression of increased formation of autolysosomes which are required to complete the process of autophagy (**F**). Additionally mRNA levels of PDK4 was evaluated (**G**). Magnification ×30k. Results expressed as mean ± S.D., *P < 0.05, **P < 0.01, ***P < 0.001.

To evaluate the autophagy by the meaning of autolysosomes formation, we performed immunofluorescence staining for LAMP2 (Fig. 10A). Based on the confocal photographs, relative fluorescence intensity of LAMP2 was evaluated (Fig. 10B). Obtained data indicates increased levels of LAMP2 in ASC_EMS_. Moreover, visualization of mitochondrial network revealed formation of long, interconnected tubular network in control group. On the contrary, ASC_EMS_ mitochondria were characterized as a round, separate tubules (Fig. 10A). Merged photographs of MitoRed and LAMP2 revealed increased mitophagosomes formation in ASC_EMS_. Additionally, flow cytometer for LAMP2 was performed (Fig. 10C), and confirmed the results of immunofluorescence staining as we observed increased amount of LAMP2 in ASC_EMS_. Moreover, the expression of p62 was significantly decreased in ASC_EMS_ (*P* < 0.01) (Fig. 10D). Growing evidence supports that autophagy has an important protective role in resistance to stress or injury as well as in differentiation process. To determine the intensity of that process in ASCs we used TEM technique (Fig. 11A and B). Obtained results revealed increased formation of autophagosomes and autolysosomes in ASC_EMS_. To evaluate mitochondrial biogenesis we evaluated mRNA levels of PGC1 α (Fig. 11C). ASC_EMS_ were characterized by decreased PGC1 α expression in control (*P* < 0.05) and osteogenic conditions (*P* < 0.001). Interestingly, during osteogenesis PGC1 α expression increases in both investigated groups, which indicates on enhanced mitochondrial biogenesis during differentiation. Interestingly, autophagic clearance of impaired mitochondria during osteogenesis seemed to be decreased in ASC_EMS_ as the expression of Parkin (Fig. 11D) was significantly reduced in those cells in comparison to control (*P* < 0.001). No statistical significance was observed in Parkin expression among investigated groups cultured under control conditions. Moreover, we evaluated the expression of two autophagic markers Beclin 1 and LC‐3. Beclin 1 is involved in the early phase of autophagosome formation, whereas LC‐3 serves as a typical marker of competed autophagosomes as it is associated with its membranes. The ratio of LC‐3 and Beclin (Fig. 11E) indicates on increased formation of autophagosomes during osteogenesis process in both groups with the increased intensity in ASC_EMS_ (*P* < 0.01). As complete process of autophagy requires the formation of autolysosomes, we determined the expression of LAMP‐2 (Fig. 11F). Similarly, the expression of LAMP‐2 increases during osteogenesis in both groups, but it was significantly higher in ASC_EMS_ in comparison to control group (*P* < 0.05). Even under control conditions the expression of LAMP‐2 was increased in ASC_EMS_ (*P* < 0.05). Additionally, we investigated mRNA level of PDK4 (Fig. 11G). It expression was decreased in ASC_EMS_ in both, control and osteogenic conditions (*P* < 0.001), which indicates increased metabolism and ATP production by the oxidative phosphorylation process.

### The expression of HIF‐1‐α and FOXO1

**Figure 12:**
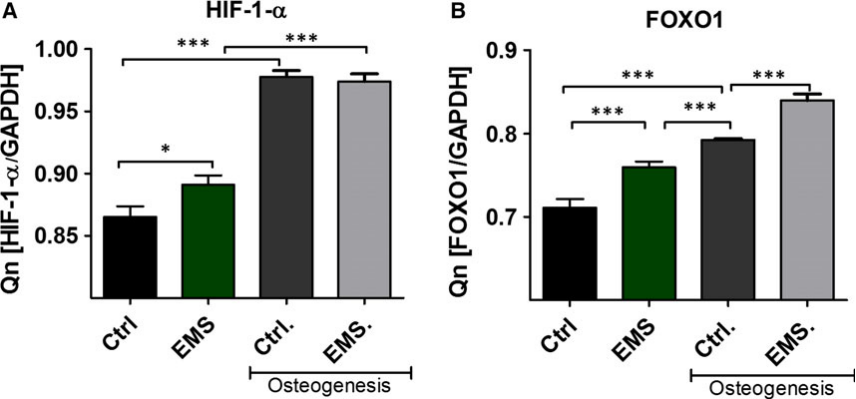
The expression of HIF‐1‐α and FOXO1. Using RT‐PCR the mean mRNA levels of HIF‐1‐α (**A**) and FOXO1 (**B**) was established. The expression of both transcripts increases during osteogenic differentiation of ASCs. *P < 0.05, ***P < 0.001.

Using RT‐PCR the mean mRNA levels of Hypoxia Induced Factor (HIF)‐1‐α (Fig. 12A) and FOXO1 (Fig. 12B) was established. The expression of both transcripts increased during osteogenesis in ASC_EMS_. In control culture HIF‐1‐α was increased in ASC_EMS_ (*P* < 0.05) while there was no differences in its amount during osteogenesis. Similarly, the expression of FOXO1 in control condition was increased in ASC_EMS_ (*P* < 0.001). Moreover, it was also increased during osteogenic stimulation in ASC_EMS_ (*P* < 0.001) as well.

## Discussion

Recently, more and more attention has been paid to investigate EMS, its clinical, social and economic consequences. EMS, increasingly frequent multifactorial disease in horses, is characterized by pathological obesity, hyperglycaemia, hyperlipidaemia 7. Moreover, growing evidence indicates on causative link between adipose tissue inflammation and the development of insulin resistance in equines. Recently, cellular therapies including application of stem progenitor cells hold great promise in the treatment of diabetes type 2 in humans 10. In turn, in equine veterinary medicine practice, stem progenitor cells are widely used, and these type of therapies are well accepted in veterinary society 31, 32, 33. However, the cytophysiological conditions of progenitor cells, used for engraftment seems to be fundamental factor, that might determine the effectiveness of clinical therapy. In this report we showed, that ASC isolated form EMS horses suffer for: reduced multipotency and clonogenic potential, elevated senescence, increased DNA methylation status and accumulation of oxidative stress factors. Moreover, those cells were characterized by partially impaired osteogenic differentiation potential. These facts, in consequence might be a limited factor, when application of ASCs isolated from EMS horses (ASC_EMS_) in cellular therapy is considered.

Here, we have found, that ASC_EMS_, among other surface markers, that are typical for MSCs including CD105^+^, CD90^+^ and CD45^−^ exhibited significantly higher expression of CD44^+^ when compared to ASC isolated from healthy, non‐obese horses. CD44 a cell‐surface glycoprotein is an immune cell receptor, which is involved in activation of inflammatory cells. The research conducted by Kodama and colleges 47, 48 showed, that CD44^+^ is significantly upregulated, however in white adipose tissue of obese, diabetic mice as well as humans. Multiple studies have shown, that CD44^+^ knockout mice fed high fat diet, did not developed signs of obesity and/or diabetes type II 49. It is well known, that up‐regulation of CD44^+^ stimulates and activates inflammatory cell migration and infiltration that finally leads to further increasing of inflammatory reaction in adipose tissue 49. These stands in good agreement with our previous finding where we have showed, that adipose tissue of EMS Welsh ponies are abundantly infiltrated by macrophages and other inflammatory cells population, that secrete an elevated pro inflammatory cytokines including IL‐1, IL‐6 and TNF‐alpha 6.

Due to decreased insulin response in sensitive tissues combined with excess glucose accumulation, EMS leads to chronic hyperglycaemia and hyperinsulinaemia. Those unfavourable environment, negatively affects the ASC population which resides within the adipose tissue. It was shown that insulin resistance and type 2 diabetes (T2D) caused alternations in mitochondria metabolism. The patients with T2D have shown decreased mitochondrial density and biogenesis 50, 51. Deteriorated mitochondria produce excessive amount of ROS which in consequence leads to protein an mitochondrial DNA damage causing cell death 52, 53.

In this study, using TEM, we observed a high frequency of mitochondrial morphological abnormalities in ASC_EMS_, including disarrayed cristae and vacuoles formation. Moreover, ASC_EMS_ were characterized by increased ROS production in comparison to control group. Furthermore, we evaluated whether this mitochondrial impairments are responsible for the augment of apoptosis in ASC_EMS_. We found that, cytochrome c is released from mitochondria to the cytoplasm suggesting activation of intrinsic apoptotic pathway in those cells. Moreover, we observed up‐regulation of p21 and decreased ratio of Bcl‐2/BAX followed by increased number of TUNEL positive cells in ASC_EMS_ which confirmed that hypothesis. Taking into consideration above mentioned facts, increased apoptosis is responsible for decreased proliferation rate and clonogenic potential observed in ASC_EMS._

The controlled regulation of mitochondrial abundance through biogenesis or degradation is critical for proper cell function and bioenergetics status. Forni *et al*. 54 showed that murine MSC commitment to differentiation is regulated by mitochondrial dynamics including processes of fission/fusion. The study conducted by Mandal *et al*. 55 showed that proper mitochondrial function is essential for proliferation of undifferentiated ESCs. The elimination of dysfunctional mitochondria with low membrane potential in mammalian cells is mediated by the Parkin protein. Parkin is selectively recruited to dysfunctional mitochondria with low membrane potential in mammalian cells 56. Its loss is associated with swollen mitochondria and muscle degeneration in Drosophila melanogaster, as well as mitochondrial dysfunction and increased susceptibility to mitochondrial toxins in other species [57, 58, 59.]. Parkin mediates the engulfment of mitochondria by autophagosomes and thereby the selective elimination of impaired mitochondria occurs 60. It was also proved that mitophagy is implicated in the regulation of quality control, number and distribution of mitochondria during hematopoietic stem cells maintenance and differentiation 61. On the other hand, mitochondrial biogenesis is a complex process involving the coordinate expression of mitochondrial and nuclear genes 62. It was shown that, PGC α regulates mitochondrial biogenesis by serving as a coactivator of multiple transcription factors 63. However, the relation of autophagy and mitochondrial biogenesis in ASC remains poorly understood. Our RT‐PCR results showed that both, PGC α and Parkin expression was significantly decreased in ASC_EMS_ This fact may contribute to deteriorated osteogenic differentiation of ASC_EMS_ as it was shown, that shift towards a more oxidative phenotype and increased mitochondrial mass is required to initiate that process 64, 65. Here, we have found, that ASC_EMS_, in comparison to healthy donors ASCs, secreted abundantly more MV's rich in tumour suppressor p53 (p53). These observations indicate on partially lost immunomodulatory effect of ASC_EMS_ that might seriously limit their clinical application. The elevated secretion of p53 might result from accumulation of oxidative stress factors in ASC_EMS_ and pushed them in more apoptotic neither vital nature. Here we have observed, that ASC_EMS_ accumulated significantly more ROS, when compared to ASC isolated from healthy donors, what might explained their reduced proliferative activity, clonogenic potential and prolonged population doubling time. What is more, we observed that Oct‐4, but not Sox‐2 and NANOG, were significantly down regulated in ASC_EMS_ when compared to ASC obtained from healthy donors. This stands in contrary with Dentelli and colleges findings 66, that have found, however in human ASC derived from visceral adipose tissue up‐regulation of Oct4 transcripts in diabetic patients when compared to healthy donors. Oct4 is known as a critical transcription factor involved in maintaining ‘stemness’. Its activity is dependent on many environmental as well as physiological factors including DNA methylation, oxidative stress, senescence and ageing 67. Although, we observed significant downregulation of Oct4 mRNA level, FOXO1 that is recognized as a tight controller of pluripotency in embryonic stem cells, where up regulated in both native ASC_EMS_ as well as in osteoblast precursor. FOXO1 belongs to proteins that regulates insulin signalling, gluconeogenesis, insulin resistance and immune cell migration 68. It is also involved in controlling cell fate through oxidative stress, apoptosis and/or autophagy that finally pushed the cells either in ‘regenerative’ or ‘demise’ direction 69. The observed here, up‐regulation of FOXO1 in ASC_EMS_ might suggests, that ASC_EMS_ partially maintain their pluripotency and our hypothesis is, that through the entrance into autophagy process they might try to defend their pluripotency and survive in unfavourable, pro‐inflammatory micro‐environment of adipose tissue.

Additionally, it was reported, that diabetes conditions, through oxidative stress impairs mitochondrial functionality and activity 70. This seriously reduce the energetic status of cells and finally might lead to disturbances in their differentiation potential. Here, we have showed, that together with FOXO1 up‐regulation, the following transcripts PGC1α, PARKIN and PDK4, that regulates mitochondrial biogenesis, dynamics and mechanistic were significantly downregulated in ASC_EMS_. It might be due to accumulation of mitochondrial ROS, observed in ASC_EMS_ as well as because the ultrastructural changes in mitochondria, that disrupt their function. Moreover, the activity of mitochondria in ASC_EMS_ were significantly reduced when compared to healthy ASC. Thus, we concluded, that ASC_EMS_ are marked with elevated cytosolic and mitochondrial ROS, that disables a part of mitochondria from their normal functions. Next, we investigated if induction of autophagy and/or mitophagy might be a protective mechanism of ASC_EMS_ against apoptosis. The autophagic pathway can be activated under different stimuli such as starvation 71, DNA damage and ROS 72, 73, thereby eliciting a cytoprotective response in cells, that helps to overcome stressful situations. Thus we speculate, that impaired mitochondria in ASC_EMS_ leads to decreased ATP production in those cells triggering ‘autophagic turnover’. Considering that autophagy yields cell metabolic precursors that can be used for ATP generation or protein synthesis 74, it is tempting to speculate that autophagy helps those cells to undergo differentiation process. We observed advantage of LC‐3 over Beclin expression (LC‐3/Beclin), that indicate on creation of autophagosomes in ASC_EMS_ in comparison to the ASC of healthy individuals. LC3 is widely used to monitor autophagy and serves as typical marker for creation a completed autophagosomes. Moreover, we also observed up‐regulation of LAMP‐2 expression in ASC_EMS_ that suggests successful maturation of both autophagosomes and phagosomes. LAMP‐2 has also been described as a receptor for the selective degradation of cytosolic proteins in the lysosome or chaperone‐mediated autophagy. It was shown, p62 accumulates when autophagy is inhibited, and decreased levels are observed when autophagy is induced. Thus, p62 may be used as a marker to study autophagic flux. In our study, we observed decreased expression of p62 in ASC_EMS_, which stands with good agreement with our other observations, indicating that increased autophagy may be cytoprotective mechanism that those cells use to survive in the inflammatory microenvironment of adipose tissue. Moreover, it may results from decreased energy production in those cells. Finally, TEM technique allowed us to visualize advantages of mito ‐ and autophagasome formation in ASC_EMS_ when compared with healthy ASC. Our data stand with a good agreement with Nuschke *et al*. 75 who showed that human MSCs consume accumulated autophagosomes early in differentiation confirming autophagy key role in that process. Moreover, it was described that hypoxia‐mediated differentiation of RAW264.7 depends on autophagy activated *via* HIF‐1‐α 76. Ablation of HIF‐1‐α/BNIP3 pathway prevented autophagy induction, as well as hypoxia‐induced ontogenesis. It strongly correlates with our data, as we observed increased HIF‐1‐α expression in both native and osteogenically differentiated ASC_EMS_. Those basal up‐regulation of HIF‐1‐α may results from increased insulin levels in circulating blood in EMS horses. Interestingly, Aymard *et al*. 77, noticed that increase in LC3 participate in keratinocyte differentiation and autophagy regulation. Moreover, they suggested an increase in lysosome‐dependent degradation activity during this process. In our study, we also discovered elevated expression of LC3 during differentiation, interestingly significantly higher in ASC_EMS_ group.

Mitophagy, as a mechanism, that protects against apoptosis, is induced in EMS horses by accumulation of ROS that is destroying mitochondrial lipids, DNA and proteins that finally leads to releasing Ca^2+^ and cytochrome c. We have observed abundant accumulation of cytochrome c in ASC_EMS_ when compared to healthy ASC. These explained observed BCL‐2/BAX downregulation of ASC_EMS_ with simultaneous up‐regulation of p21, that together indicate of advantages of apoptotic nature of ASC_EMS_ over healthy donor cells. Moreover, the p21 up‐regulation may be responsible not only for the execution of cell cycle arrest in ASC_EMS_, but also for increased apoptosis as it plays a crucial role in mediating cellular senescence. We speculate, that immunologically and physiologically weakened ASC_EMS_ are touched by the oxidative stress factors, including accumulated ROS and nitric oxide, which negatively affect the formation of extracellular matrix of osteoblast precursor. We have found, that osteoblast precursors derived from ASC_EMS_ exhibited significant decreased in proliferative activity and formed less osteogenic nodules when compared with healthy progenitor cells. It might be due to elevated accumulation of ROS during differentiation process. Interestingly SOD, was synthesized on comparable level in ASC_EMS_ when compared to the control cells. These shed a positive light on anti‐oxidative properties of ASC_EMS_ during osteogenic differentiation, that as we suspects are positive consequences of previously inducted auto/mitophagy in native, non‐differentiated ASC_EMS_. Our data stands with the good agreement with Chen *et al*. 65, who also observed increased anti‐oxidative protection during osteogenic differentiation. In the course of the osteogenic differentiation process, we have found, that expression of bone BMP‐2, as well asColl‐1 on mRNA level was significantly downregulated in ASC_EMS_, whereas expression of RUNX2, OCN and ALP were comparable between both groups. BMP‐2 plays an important role in cell proliferation, differentiation, skeletal development and bone formation 78. It was shown, that in particular BMP‐2 is involved in differentiation process on all following steps: differentiation period, bone formation period and remodelling period. The impairment of BMP‐2 and Col‐1 expression in ASC_EMS_ might be related with diabetes condition in EMS horses, since it was shown that human obese patients with diabetes tend to have an increased risk of osteoporosis or bone fractures that may be related to hyperglycaemia 79. Moreover, hyperglycaemia or hyperlipidaemia are the factors, that impairs the osteogenic differentiation potential of MSC. However recently, FOXO1‐dependent oxidative defense was reported to be important for bone formation and bone mass homeostasis 80. Interestingly, FOXO1 inhibit osteoblast apoptosis through the suppression of oxidative stress 81. The observed here downregulation of BCL‐2 in ASC_EMS_ simultaneously with up‐regulation of FOXO1 might maintain osteoblast differentiation. However, the downregulation of PGC1α, PARKIN and PDK4 in differentiated ASC_EMS,_ that indicates on mitochondrial biogenesis and function deteriorations, might partially explained downregulation of BMP‐2 as well as Col‐1.

Moreover, we evaluated the DNA methylation status of isolated ASC as hypermethylation is known to be associated with ageing and diseases progression 82. As it was shown by Zhao *et al*. 83 global DNA methylation is associated with insulin resistance. Additionally, Kim *et al*. reported that Obesity‐induced DNA hypermethylation of the adiponectin gene mediates insulin resistance 84. Moreover, it was shown that chronic inflammation causes aberrant DNA methylation 85. Here, we speculate that unfavourable microenvironment in which ASC_EMS_ reside, may increase the amount of 5‐mC leading to decreased proliferative and secretory activity of those cells.

Data have indicated that ASC isolated from horses, might be used safety in cellular therapy. However, EMS impairs ASC's self‐renewal and differentiation properties. Here, we provided evidence, that ASC_EMS_ induced mitophagy to be able to survive in unfavourable microenvironment of adipose tissue and when differentiated *in vitro* into osteoblast, exhibited partially reduced osteogenic potential. Hence, application of ASC_EMS_ into endocrinological or ortophedical practice requires further investigation and analysis in the context of safeness of their application.

## Conclusion

In brief, disturbances in mitochondrial morphology and dynamics, leads to alternations in bioenergetics profile during osteogenic differentiation of ASC_EMS_ resulting in their decreased proliferation rate and reduced expression of osteogenic markers BMP‐2 and collagen type I. The impaired remodelling of mitochondrial network caused by decreased biogenesis and mitophagy results in the accumulation of damaged mitochondria and excessive ROS levels. Depletion of ATP production caused by impaired mitochondria strongly affects cellular metabolism and ability to proliferate. We speculate, that those circumstances triggers autophagic turnover in ASC_EMS_ as an alternative way to generate ATP and amino acids required to increased protein synthesis during differentiation.

Our results confirmed a key role of autophagy in differentiation process in ASC and presents potential utility for autophagy modulation in optimizing MSC differentiation, which may lead to improved therapeutic effect of engrafted cells.

## Conflict of interest

The authors confirm that there are no conflicts of interest.
